# MicroRNAs from the same precursor have different targeting properties

**DOI:** 10.1186/1758-907X-3-8

**Published:** 2012-09-27

**Authors:** Antonio Marco, Jamie I MacPherson, Matthew Ronshaugen, Sam Griffiths-Jones

**Affiliations:** 1 Faculty of Life Sciences, Michael Smith Building, Oxford Road, University of Manchester, Manchester M13 9PT, UK

**Keywords:** Arm switching, Gene regulation, miRNA, Target prediction

## Abstract

**Background:**

The processing of a microRNA results in an intermediate duplex of two potential mature products that derive from the two arms (5′ and 3′) of the precursor hairpin. It is often suggested that one of the sequences is degraded and the other is incorporated into the RNA-induced silencing complex. However, both precursor arms may give rise to functional levels of mature microRNA and the dominant product may change from species to species, from tissue to tissue, or between developmental stages. Therefore, both arms of the precursor have the potential to produce functional mature microRNAs.

**Results:**

We have investigated the relationship between predicted mRNA targets of mature sequences derived from the 5′ and 3′ arms of the same pre-microRNAs. Using six state-of-the-art target prediction algorithms, we find that 5′/3′ microRNA pairs target different sites in 3′ untranslated regions of mRNAs. We also find that these pairs do not generally target overlapping sets of genes, or functionally related genes.

**Conclusions:**

We show that alternative mature products produced from the same precursor microRNAs have different targeting properties and therefore different biological functions. These data strongly suggest that developmental or evolutionary changes in arm choice will have significant functional consequences.

## Background

MicroRNAs are crucial regulators of gene expression whose biogenesis is tightly controlled by multiple enzymes [1,2]. Primary microRNA transcripts are single-stranded RNA molecules that fold into hairpins, and are cleaved by two RNases producing an approximately 22-nucleotide RNA duplex [1]. In a process called arm-sorting or strand-sorting, one of the sequences of the duplex (derived from one of the arms of the precursor hairpin) associates with the RNA-induced silencing complex (RISC), which will mediate mRNA translational repression or transcript degradation (reviewed in [1]). The other arm (the star sequence or microRNA*) is generally considered a byproduct and is typically degraded [3]. However, it has become clear that both arms of the hairpin may produce functional mature products in many cases [4,5]. Indeed, microRNA* sequences are often highly expressed, evolutionarily conserved, and associated with RISC proteins [5]. For example, the microRNA* product of the *Drosophila bantam* gene is present at approximately 10-fold greater levels than any other microRNA product across a range of cell types and developmental stages. MicroRNA* sequences have been shown to be loaded into the RISC complex and to repress target genes both *in vivo* and *in vitro*[5-7].

Recent studies have shown that precursor microRNAs can change the arm from which the dominant functional mature microRNA is produced. This process, called arm-switching, occurs both in different tissues and developmental stages [4,8,9] and during evolution [10-13]. During microRNA biogenesis, both arms are produced at equal amounts in a given cell and, later on, one of the arms is usually degraded. For that reason, one may expect that microRNAs from the same precursor have similar targeting properties. Alternatively, since opposite arms of the hairpin have different sequences, it is expected that they target different sites. If these sites are in different transcripts, changes in arm usage would have the potential to alter microRNA function. So far, the only studied case is the mir-100/10 family, for which we have shown that opposite arms of precursor microRNAs do not significantly share target genes [13]. The functional consequences of changes in arm usage have not been extensively studied.

MicroRNA target recognition is mediated by complementary base-pairing between the microRNA and the 3′ untranslated regions (UTR) of targeted transcripts [14]. The number of experimentally validated microRNA/target pairs remains limited. However, computational prediction of microRNA targets has been widely used, although these approaches produce high rates of false positives [15]. In spite of this limitation, computational prediction of targets permits the study of general binding properties of a given microRNA. A widely accepted view of microRNA target preferences relies on nucleotides 2 to 7 of a microRNA, the so-called seed sequence, which recognizes binding sites often by perfect complementarity to the targeted transcripts (reviewed in [14]). However, distinct modes of target recognition have been described and they form the basis of distinct prediction algorithms. Since different prediction strategies are based on different assumptions and may give quite different results, it is often useful to apply a variety of algorithms to study the targeting properties of microRNAs.

**Figure 1:**
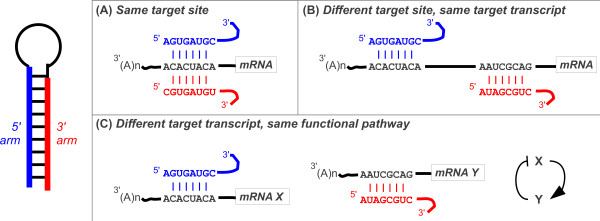
**Possible targeting properties of 5′/3′ microRNA pairs.** (**A**) Both 5′ and 3′ products bind to the same target. (**B**) MicroRNA products bind to different sites in the same transcript. (**C**) MicroRNA products bind to different transcripts that act in the same functional pathway.

Here, we use multiple target prediction algorithms to predict targets of human and fly microRNAs. We assess whether pairs of mature sequences derived from the 5′ and 3′ arms of the same precursor target identical sites (Figure 1A), different sites in the same gene transcripts (Figure 1B) and different genes in the same functional pathways (Figure 1C).

## Results

### Mature microRNAs from the same precursor have distinct target sites

We tested whether alternative mature microRNA products derived from the 5′ and 3′ arms of the same precursor share predicted target sites (Figure 1A). We predicted all canonical seed targets for all microRNAs in *Drosophila melanogaster* and human [14] and counted how many target sites have pairs of microRNAs from the same precursor in common. We observed that not a single predicted site was shared between the pairs of mature microRNAs from *Drosophila*. In humans, only one 5′/3′ microRNA pair, derived from mir-3648, had common targets, sharing 61 predicted sites out of a total of 569 and 455 sites predicted for the 5′ and 3′ microRNAs respectively. This is explained by the fact that both mature sequences are GC rich, and both seed sixmers are identical: GCCGCG. A closer inspection of the patterns of deep sequencing reads mapped to the *mir-3648* locus (as shown in miRBase; [16]) suggests that mir-3648 may not be a *bona fide* microRNA, since it does not show a read pattern compatible with small RNA processing. In general, mature microRNAs from opposite arms have different sequences, therefore their propensity to target different sites is expected.

### 5′/3′ microRNA pairs target non-overlapping gene lists

UTRs may contain multiple target sites for different microRNAs. Therefore 5′/3′ pairs of microRNAs may target sites in the same transcript (Figure 1B). To test whether 5′/3′ microRNA pairs target common genes, we predicted regulated genes using six different and complementary methods: canonical seeds, miRanda, PITA, Diana-microT, RNAhybrid and TargetScan (with conservation - see Methods). For each 5′/3′ microRNA pair we compared the overlap between the predicted target lists and the expected overlap for random pairs of microRNAs (see Methods).

**Figure 2:**
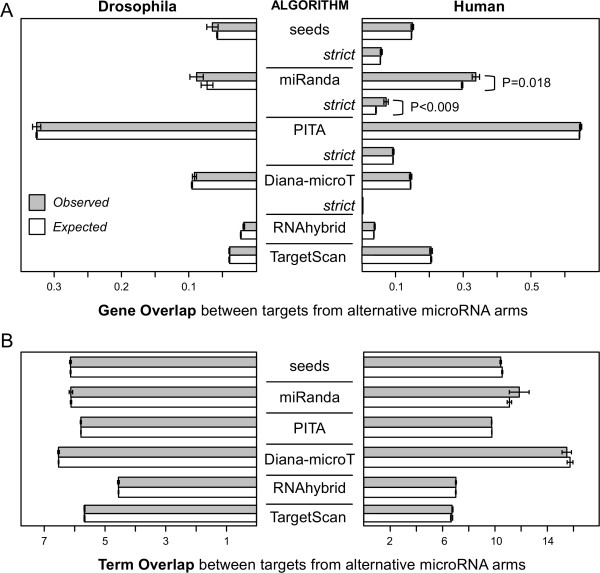
**Distribution of target overlaps between 5′/3′ microRNA pairs.** Average overlap of target predictions for multiple algorithms (grey boxes) and expected overlap based on random sampling (white boxes). Error bars depict standard errors of the means. Statistical differences between distributions underlying the plotted data were assessed by one-tailed Kolmogorov-Smirnov tests with Bonferroni correction. Only *P*-values below 0.05 are shown. (**A**) Overlap between lists of targeted genes from microRNA pairs from the same precursor. The human datasets include additional values for strict sets of predictions for four of the algorithms. (**B**) Term overlap between the targets of 5′/3′ pairs of microRNAs. Human term overlap values were calculated, when available, for the *strict* target prediction sets.

Canonical seeds, PITA, Diana-microT, RNAhybrid and TargetScan methods consistently showed that the overlap between genes targeted by 5′/3′ microRNA pairs is not statistically different from random expectation (Figure 2A). Only the miRanda algorithm suggests a significant overlap of genes targeted by 5′/3′ microRNA pairs (see below). The number of microRNA pairs with overlapping target gene predictions in the human dataset is about twice that for *Drosophila*. This is likely due to the fact that human 3′ UTRs are longer than those from *Drosophila*, and therefore the number of microRNAs predicted to target each transcript is significantly larger. Therefore, we performed a second analysis in the human set using a more stringent set of parameters (see Methods). These *strict* predictions yielded smaller overlapping values, but the overall findings remain robust to the parameter changes: only the miRanda set showed significant differences between the observed and the expected overlap values (Figure 2A).

We investigated whether the observed overlap for miRanda predictions of gene targets of 5′/3′ microRNAs pairs could be explained by sequence composition biases. In particular, programs that use hybrid stability to detect microRNA targets (such as miRanda) may be biased by variable GC content [17]. We therefore studied the potential effect of composition bias on predicted microRNA targets in humans. We find that the number of predicted gene targets is highly correlated with the GC content of the microRNA (R^2^ = 0.72, *P* <0.001). There is also a positive correlation between the microRNA duplex GC content and the overlap between the genes targeted (R^2^ = 0.58, *P* <0.001). After removing those microRNAs with high GC content (defined as greater than 67% as in [17]), the overlap between target genes of human 5′/3′ microRNA pairs was still significant (*P* = 0.003). The overlap between miRanda predictions for 5′/3′ microRNAs pairs is therefore robust to sequence bias.

### 5′/3′ microRNA pairs do not target genes in the same functional classes

Different genes targeted by different microRNAs may have related functions or be involved in related pathways (Figure 1C). The functional similarity of two genes can be quantified by assessing the similarity of their annotation, for example using Gene Ontology (GO) terms [18,19]. This class of methods is known as semantic similarity measures. Semantic similarity using GO term annotation has been widely applied in genomics to compare functional similarity between pairs of genes (for example, [19,20]). Here we use a measure called average term overlap (TO) to estimate the functional similarity between lists of genes (see Methods). Values for average TO were calculated for the lists of genes targeted by 5′/3′ pairs of microRNAs. We did not observe any significant overlap in the functions of genes targeted by 5′/3′ pairs of microRNAs based on GO annotations with any of the algorithms. A slight bias (although not significant) for 5′/3′ microRNAs to target genes with related functions using miRanda (Figure 2B) is explained by the significant overlap of targeted genes discussed above (Figure 2A). From these analyses, we conclude that alternative microRNAs from the same precursor have significantly different targeting properties.

### Cases in which 5′/3′ pairs have similar targets

**Figure 3:**
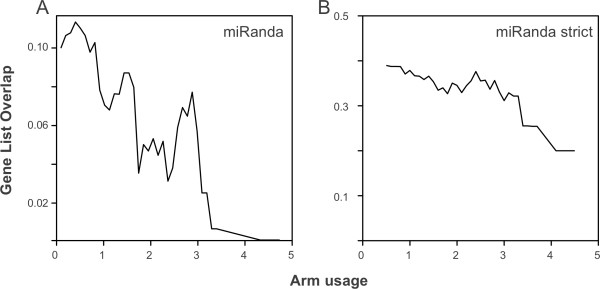
**Effect of microRNA arm usage on targeting properties.** Sliding-window plot showing (**A**) mean target list overlap and (**B**) mean term (function) overlap with respect to the microRNA arm usage bias (see Methods).

We have shown that miRanda predictions suggest that some 5′/3′ microRNA pairs tend to target common genes. We explored whether the relative amount of microRNA produced from each arm of the hairpin precursor is associated with the targeting properties for the human dataset. In Figure 3 we plot the average gene overlap for different levels of arm usage bias. Arm usage bias reflects the number of reads from deep sequencing experiments that map to one arm with respect to the other (see Methods), and was calculated only for microRNAs that have reads associated with both arms. The impact of arm usage bias in the targeting properties of human microRNAs is shown in Table 1. Where pairs of alternate microRNAs from the same hairpin are produced at ratios of at least 10:1 (that is, a mature product from one arm dominates), we find that the 5′/3′ pairs of microRNAs do not bind to overlapping lists of genes. MicroRNAs with low or no arm usage bias produce pairs of mature sequences that do bind to overlapping lists of genes (Table 1). By contrast, mature 5′/3′ microRNA pairs that are expressed at similar levels tend to bind more similar lists of genes (Figure 3). We observe a similar pattern in *Drosophila*: the subset of microRNAs producing mature sequences approximately equally from both arms share more targets than expected by chance (Table 1), although the differences are not statistically significant. The set of human microRNAs that produce similar amounts of mature products from each arm (ratio less than 3:1, and a minimum of 10 reads mapping to either arm) is shown in Table 2. Three out of the 11 pairs have a target overlap above the expected value (>0.071). We therefore show that the significant overlap of predicted gene targets of 5′/3′ microRNA pairs can be attributed to microRNAs that produce approximately equal amounts of mature sequences from both arms.

**Table 1:** **Effect of arm usage bias on gene overlap of miRanda predictions of 5**^**′**^**/3**^**′**^**microRNA pairs**

		**Low or no arm usage bias**	**High arm usage bias**^**b**^
*Drosophila*			
	Observed (SEM)	0.1026 (0.0179)	0.0823 (0.0122)
	Expected (SEM)	0.0840 (0.0011)	0.0706 (0.0009)
	*P-value*^*a*^*(N)*	*0.183 (62)*	*0.422 (96)*
Human			
	Observed (SEM)	0.3444 (0.0248)	0.3143 (0.0214)
	Expected (SEM)	0.2906 (0.0021)	0.3104 (0.0019)
	*P-value*^*a*^*(N)*	*0.059 (87)*	*0.5369 (70)*
Human (strict set)			
	Observed (SEM)	0.0769 (0.0152)	0.0361 (0.0846)
	Expected (SEM)	0.0464 (0.0012)	0.0430 (0.0011)
	*P-value*^*a*^*(N)*	*0.028 (87)*	*0.269 (70)*

^a^*P-values* for differences between the expected and observed distributions calculated with one-tailed Kolmogorov-Smirnov non-parametric test. ^b^High arm usage bias microRNAs defined as those with a ratio of reads mapping to each arm of at least 10:1. SEM: standard error of the mean.

**Table 2:** Human microRNAs with low arm usage bias

**MicroRNA**	**5′ targets**	**3′ targets**	**Common targets**	**Gene overlap**	**5′ reads**	**3′ reads**	**Arm usage**
mir-378	1,988	55	53	0.026	13	10	0.11
mir-32	0	0	0	0	15	11	0.13
mir-3648	170	2,926	170	0.055	9	13	0.16
mir-128-1	1,695	9	9	0.005	25	17	0.17
mir-193a	1,470	175	155	0.094	284	192	0.17
mir-187	468	369	175	0.209	12	19	0.20
mir-183	0	0	0	0	29	18	0.21
mir-500a	428	307	184	0.250	16	9	0.25
mir-361	30	801	27	0.032	46	25	0.27
mir-106b	1	605	0	0	1,394	724	0.29
mir-424	0	0	0	0	167	84	0.30

Targets predicted with miRanda, with a score threshold of 1,000.

## Discussion

In this work, we have shown that, in general, 5′/3′ mature microRNA sequences derived from the same microRNA precursor target non-overlapping lists of genes. The only exceptions derive from predictions made with the miRanda algorithm [21] of targets of mature sequences produced in equal concentrations from both arms of the precursor. miRanda takes into account hybrid stability of the target and the microRNA, as well as strong sequence complementarity in the seed region [21]. We envisage two possible explanations for the different result from miRanda predictions. On the one hand, the relaxation of the requirement for perfect complementarity in the seed region may allow miRanda to detect targets and trends that escape other prediction algorithms (probably at the expense of prediction specificity). Indeed, a small number of cases of 5′/3′ microRNA pairs binding to the same transcript have been described (for example, [22]). On the other hand, miRanda predictions may be susceptible to unknown biases such that the observed pattern is an artifact of the algorithm (although we rule out the effects of GC bias here). Nevertheless, all six different algorithms with two different sets of parameters, covering the spectrum of most existing target prediction algorithms [23], concur that 5′/3′ mature microRNA pairs do not target the same genes or pathways when the precursor produces functional products primarily from one of the arms.

Early experiments suggested that the thermodynamic properties of the microRNA duplex determine the sequence that is incorporated into the RISC, and hence, which arm is functional [5,6,24]. However, we recently proved that identical duplex sequences in *Drosophila melanogaster* and the beetle *Tribolium castaneum* can produce functional microRNAs from opposite arms [13]. Moreover, the dominant arm can change within the same species in different developmental stages or tissues [9-11]. This suggests that arm sorting can be determined by signals outside the mature microRNA duplex. Thus, changes in arm usage may occur without changing the nucleotide sequences of mature microRNAs, such that the potential targeting properties of both arms are unchanged (see also [5,6]). We have described five instances of arm switching between *Drosophila* and *Tribolium* microRNAs [12]: mir-10, mir-33, mir-275, mir-929 and mir-993. These microRNAs are highly expressed and, in each case, mature sequences are produced in ratios of around 10:1 [25]. In this work, we provide evidence that the targeting properties of 5′/3′ microRNA products are not similar when one mature product dominates. Therefore, arm-switching events in these five microRNAs [12] are predicted to lead to functional changes, as we previously suggested for mir-10 in *Drosophila* and *Tribolium*[13].

## Conclusions

Alternative mature products from the same precursor microRNA have different targeting properties. Exceptions to this rule are observed for microRNAs from which both arms produce significant amounts of mature products using miRanda gene predictions. We therefore strongly suggest that microRNA arm preferences have important functional consequences. Comparative analysis of regulatory networks accounting for microRNA arm usage will be slightly more complex, yet biologically more meaningful.

## Methods

We extracted all fly (*D. melanogaster*) and human (*Homo sapiens*) microRNAs from miRBase (version 16; [16]). This version of miRBase does not index 5′ and 3′ mature sequences for all microRNAs. Where a single mature sequence from a microRNA precursor is reported, we selected as the miR* sequence the most abundant read from the appropriate arm from high-throughput sequencing data displayed in miRBase (December 2010; [16]) and discarded sequences with no evidence for a miR* sequence. This resulted in a total of 163 and 426 pre-microRNAs in fly and human respectively. The expression datasets used in this analysis are listed in Additional file 1: Table S1.

We used six different algorithms to detect potential targets of mature microRNA sequences: canonical seeds as described in [14]; miRanda [21], a method based on hybrid energy and stability; PITA [26], which takes into account the site accessibility at 3′ UTRs; Diana-microT [27], a predictor that combines multiple features; RNAhybrid [28], which detects stable RNA-RNA duplexes; and TargetScan [29,30], a canonical seed detection program that also takes into account conservation of microRNAs and target sites. We ran TargetScan to identify target sites conserved in at least two species in the 3′ UTR alignments available from their webpage [30]. We generated target prediction datasets for each algorithm using default parameters. We also generated a second prediction set for human microRNAs (called the *strict* set) using each algorithm with the following parameter modifications: at least two sites in canonical seed predictions; miRanda targets with a score above 1,000, to reduce the number of targets to a tenth of the original predictions; PITA-predicted targets of a size of 7 to 8, with no mismatches or wobble positions; Diana-microT predictions with an MRE score above 0.6 as suggested by the authors.

We used as potential targets the largest 3′UTR available for each gene in *Drosophila* in Flybase (genome version BDGP 5.25 [31]) and in human from ENSEMBL (assembly 60 [32]). For each pair of mature products from a precursor microRNA, we identified potential targets with all six methods, and we calculated for each method the overlap between the lists of target sites as the number of commonly targeted sites divided by the total number of sites targeted by both arms (Jaccard similarity; [33]). Similarly, the overlap between lists of target genes was calculated as the number of commonly targeted genes divided by the total number of genes targeted by both arms. The expected distributions of values were calculated by selecting 10,000 random pairs of microRNA arms and calculating the target overlap for each pair.

To assess whether two lists of genes have a similar functional annotation, we cross-compared all gene pairs between the two lists and calculated semantic similarity using the term overlap (TO) measure [19,34] for the ‘biological process’ domain of Gene Ontology [35]. Average TO values for pairs of gene lists are defined as:

$$TO=\frac{\sum_{i=1}^{n}\sum_{j=1}^{m}T\left\{G_{i},G_{j}\right\}}{nm}$$

where *T{G*_*i*_*,G*_*j*_*}* is the number of common GO terms to which genes *G*_*i*_ and *G*_*j*_ are annotated. The TO analysis in humans was performed only for the *strict* target prediction sets. Expected average TO values were calculated by generating 1,000 randomized pairs.

Arm usage is defined as the relative production of mature products from one arm with respect to the other arm, and it is calculated as described in [12]. Only microRNAs with reads in both arms were included. An arm usage of 0 means that both arms produce the same amount of product. Each unit above 0 indicates a two-fold increase in the biased production of one of the arms.

## Abbreviations

GO: gene ontology; RISC: RNA-induced silencing complex; TO: term overlap; UTR: untranslated region.

## Competing interests

The authors declare that they have no competing interests.

## Authors’ contributions

AM and SGJ conceived the project. AM and JIM performed the analyses. AM, MR and SGJ interpreted the results and wrote the manuscript. All authors read and approved the final manuscript.

## Supplementary Material

Additional file 1 **Table S1.** Gene expression datasets. Click here for file
