# Supplementary material for: MicroRNAs from the same precursor have different targeting properties
Source: Silence. 2012 Sep 27;3:8. doi: 10.1186/1758-907X-3-8 (PMC3503882; doi:10.1186/1758-907X-3-8)
Supplement: Additional file 1 — Table S1. Gene expression datasets. [file 1758-907X-3-8-S1.doc]

**Supplementary Table 1.** Gene expression datasets.

| **Species** | **GEO number** | **Tissue / stage** | **Reference** |
| --- | --- | --- | --- |
| *D. melanogaster* | GSM240749 | head | Chung et al. *Curr Biol* 2008, 18:795 |
| *D. melanogaster* | GSM272651 | S2 cell | Chung et al. *Curr Biol* 2008, 18:795 |
| *D. melanogaster* | GSM272652 | S2 cell | Chung et al. *Curr Biol* 2008, 18:795 |
| *D. melanogaster* | GSM272653 | Kc cell | Chung et al. *Curr Biol* 2008, 18:795 |
| *D. melanogaster* | GSM275691 | imaginal disc | Chung et al. *Curr Biol* 2008, 18:795 |
| *D. melanogaster* | GSM286601 | head | Chung et al. *Curr Biol* 2008, 18:795 |
| *D. melanogaster* | GSM286602 | whole body | Chung et al. *Curr Biol* 2008, 18:795 |
| *D. melanogaster* | GSM286603 | whole body | Chung et al. *Curr Biol* 2008, 18:795 |
| *D. melanogaster* | GSM286604 | embryo | Chung et al. *Curr Biol* 2008, 18:795 |
| *D. melanogaster* | GSM286605 | embryo | Chung et al. *Curr Biol* 2008, 18:795 |
| *D. melanogaster* | GSM286606 | embryo | Chung et al. *Curr Biol* 2008, 18:795 |
| *D. melanogaster* | GSM286607 | embryo | Chung et al. *Curr Biol* 2008, 18:795 |
| *D. melanogaster* | GSM286611 | embryo | Chung et al. *Curr Biol* 2008, 18:795 |
| *D. melanogaster* | GSM286613 | embryo | Chung et al. *Curr Biol* 2008, 18:795 |
| *D. melanogaster* | GSM322208 | 3rd instar larvae |  |
| *D. melanogaster* | GSM322219 | 2-4 day old pupae |  |
| *D. melanogaster* | GSM322245 | 3rd instar larvae |  |
| *D. melanogaster* | GSM322338 | 2-4 day old pupae |  |
| *D. melanogaster* | GSM322533 | adult female head |  |
| *D. melanogaster* | GSM322543 | adult male head |  |
| *D. melanogaster* | GSM360256 | 1st instar larvae |  |
| *D. melanogaster* | GSM360257 | 1st instar larvae |  |
| *D. melanogaster* | GSM360260 | 0-1 day old pupae |  |
| *D. melanogaster* | GSM360262 | 0-2 day old pupae |  |
| *D. melanogaster* | GSM364902 | 12-24hr embryo |  |
| *D. melanogaster* | GSM280082 | from 2-4 day old flies |  |
| *D. melanogaster* | GSM280083 | from 2-4 day old flies |  |
| *D. melanogaster* | GSM280084 | from 2-4 day old flies |  |
| *D. melanogaster* | GSM280085 | from 2-4 day old flies |  |
| *D. melanogaster* | GSM280086 | from 2-4 day old flies |  |
| *D. melanogaster* | GSM280087 |  |  |
| *D. melanogaster* | GSM280088 |  |  |
| *D. melanogaster* | GSM399105 | imaginal disc/brain |  |
| *D. melanogaster* | GSM399106 | female body |  |
| *D. melanogaster* | GSM399107 | male body |  |
| *D. melanogaster* | GSM371638 |  |  |
| *D. melanogaster* | GSM180328 | adult heads | Ruby et al. *Genome Res* 2007, 17:1850 |
| *D. melanogaster* | GSM180329 | adult bodies | Ruby et al. *Genome Res* 2007, 17:1850 |
| *D. melanogaster* | GSM180330 | very early embryo (0-1) | Ruby et al. *Genome Res* 2007, 17:1850 |
| *D. melanogaster* | GSM180331 | early embryo (2-6) | Ruby et al. *Genome Res* 2007, 17:1850 |
| *D. melanogaster* | GSM180332 | mid embryo (6-10) | Ruby et al. *Genome Res* 2007, 17:1850 |
| *D. melanogaster* | GSM180333 | late embryo (12-24) | Ruby et al. *Genome Res* 2007, 17:1850 |
| *D. melanogaster* | GSM180334 | larvae: 1st instar and 3rd instars | Ruby et al. *Genome Res* 2007, 17:1850 |
| *D. melanogaster* | GSM180335 |  | Ruby et al. *Genome Res* 2007, 17:1850 |
| *D. melanogaster* | GSM180336 | pupae: 0-1 day, 0-2 day, 2-4 day | Ruby et al. *Genome Res* 2007, 17:1850 |
| *D. melanogaster* | GSM180337 | tissue culture cells | Ruby et al. *Genome Res* 2007, 17:1850 |
| *H. sapiens* | GSM541796 | embryonic stem cells | Bar et al. *Stem Cells* 2008, 26:2496 |
| *H. sapiens* | GSM541797 | embryonic stem cells | Bar et al. *Stem Cells* 2008, 26:2496 |
